# Identity and Guilt as Mediators of Pro-environmental Spillover

**DOI:** 10.3389/fpsyg.2021.659483

**Published:** 2021-06-24

**Authors:** Heather Barnes Truelove, Amanda R. Carrico, Kam Leung Yeung, Jennifer M. Wolff

**Affiliations:** ^1^Department of Psychology, University of North Florida, Jacksonville, FL, United States; ^2^Environmental Studies Program, University of Colorado Boulder, Boulder, CO, United States; ^3^University of North Florida, Jacksonville, FL, United States; ^4^The Brown School, Washington University in St. Louis, St. Louis, MO, United States

**Keywords:** spillover, pro-environmental behavior, identity, guilt, licensing

## Abstract

Policymakers are interested in programs that increase targeted pro-environmental behavior (PEB) and spill over to increase non-targeted PEBs. Theoretically, guilt should lead to negative spillover and identity to positive spillover, though this has rarely been tested empirically. Additionally, little is known about how reminders of past PEB behavior might also lead to downstream spillover effects. Across two studies, participants (Study 1: 377 MTurk workers; Study 2: 172 undergraduates) were randomly assigned to write about a prior PEB, an anti-environmental behavior, or to a control condition. Subsequently, respondents were given an opportunity to perform a PEB2 and completed measures of PEB3 intentions. Results showed some evidence of positive (through increasing identity) and negative (through decreasing guilt) indirect spillover pathways from prior PEB reminders to PEB2 performance and PEB3 curtailment intentions (but not efficiency upgrade intentions). However, there were no overall spillover effects from PEB reminders to PEB2 performance or PEB3 intentions, as the positive and negative indirect effects canceled each other out. Results also showed positive spillover from PEB2 performance to PEB3 curtailment intentions through increasing environmental guilt. The strength of the spillover effects depended on the comparison group for the experimental manipulation, whether environmental guilt or global guilt was measured, and the type of PEB. The results suggest that environmental communications that remind people of their prior PEB may not meaningfully spill over to further PEB performance or intentions.

## Introduction

Public concern for the environment is high (Gallup, 2019) and as environmental problems become more dire, there is increasing urgency to reduce human impact. Though technological advancements, multi-national agreements, and economic incentives are most frequently considered as solutions, programs focusing on voluntary individual behavior change can also contribute to solving environmental problems (Steg and Vlek, 2009; Osbaldiston and Schott, 2012; Clayton et al., 2015). For example, changing climate-related behavior at the individual and household level, in the aggregate, can have considerable impact on U.S. emissions (Gardner and Stern, 2008; Vandenbergh et al., 2008; Dietz et al., 2009). Faced with limited resources, those interested in greening individual and household behavior would benefit from interventions that change not only a targeted behavior, but positively spill over to other related behaviors as well. Pro-environmental behavioral (PEB) spillover is a burgeoning area of research in conservation psychology, but many questions remain (Truelove et al., 2014; Nilsson et al., 2016; Nash et al., 2017). Among these are questions about the mechanisms that underlie spillover and how best to design interventions to generate positive spillover or avoid negative spillover.

Positive behavioral spillover occurs when the performance of one PEB increases the likelihood of future PEBs (Thøgersen, 1999; Thøgersen and Crompton, 2009; Dolan and Galizzi, 2015), while negative spillover occurs when the performance of one PEB decreases the likelihood of future PEBs (Thøgersen and Crompton, 2009). Most research has found that positive spillover is more common than negative spillover, though the likelihood of detecting positive versus negative spillover depends on how the PEBs are conceptualized (Maki et al., 2019). Several studies investigating spillover have manipulated reminders of prior PEBs as the initial PEB in the PEB spillover sequence (Van der Werff et al., 2014a,b; Lacasse, 2016; Lauren et al., 2019). In these studies, participants are led to feel that they performed many past PEBs or few past PEBs through completing checklists of behaviors with varying instructions (Van der Werff et al., 2014b; Lacasse, 2016; Lauren et al., 2019). For example, in one study, participants in the many behaviors condition were instructed to check all behaviors that they “at least sometimes do” and in the few behaviors condition participants checked behaviors they “always do” (Lauren et al., 2019). This type of procedure typically results in participants checking more PEBs in the many behavior condition than the few behavior condition, leading participants to believe that they have acted in pro-environmental ways in the past (or environmentally harmfully in the past). Other studies more directly measure behavior either by observing the initial PEB (Truelove et al., 2016) or measuring it as a self-report following an intervention (Lanzini and Thøgersen, 2014; Carrico et al., 2018; Xu et al., 2018). When the initial PEB is measured after an intervention versus manipulating perceptions of previous PEB performance, positive spillover is more common (Maki et al., 2019). Additionally, when the secondary behavior is measured as behavioral intentions versus self-reported or observed behavior, positive spillover is more likely (Maki et al., 2019). Few studies have assessed PEBs using multiple methods in the same study, which is one of the contributions of the present studies as we assess spillover effects from both PEB reminders and observed PEBs.

### Positive Spillover

Several mechanisms of positive PEB spillover have been proposed including identity and consistency effects (Truelove et al., 2014; Lacasse, 2016), self-efficacy (Lauren et al., 2016), cognitive accessibility (Sintov et al., 2017), and environmental concern (Carrico et al., 2018). The identity and consistency effects explanation has garnered the most empirical support so far (Thøgersen, 2004; Baca-Motes et al., 2013; Nilsson et al., 2016; Lauren et al., 2019). In line with self-perception theory (Bem, 1972), when people perform an initial behavior, they see themselves as the type of person who performs these types of behaviors (i.e., an environmentalist). When given an opportunity to perform additional PEBs, they are more likely to act in line with a salient identity to maintain consistency and avoid dissonance (Festinger, 1957).

Environmental self-identity, defined as viewing oneself as someone who acts in environmentally friendly ways, has specifically been implicated in PEB spillover (Whitmarsh and O’Neill, 2010; Van der Werff et al., 2014b). Several studies have found support for environmental self-identity as a mediator of the positive spillover relationship. For example, reminders of prior PEBs lead to increased environmental self-identity (Cornelissen et al., 2008; Van der Werff et al., 2014b; Lacasse, 2016), which, in turn, is related to environmentally friendly product choices (Van der Werff et al., 2014b), climate change concern and policy support (Lacasse, 2016), and PEB intentions (c.f., Truelove et al., 2016; Lauren et al., 2019). However, Van der Werff et al. (2014b, Study 4) found no spillover effect from a PEB reminder to the number of pieces of paper used in a writing task. Overall, positive indirect effects of prior PEB reminders through environmental self-identity onto various PEB outcomes have most frequently been found (Van der Werff et al., 2014a,b; Lacasse, 2016), though at least one study has found negative indirect effects onto private sphere PEBs and public sphere PEBs (Lauren et al., 2019). The picture is even less clear when the initial PEB is measured as actual PEB and not a reminder of previous PEB. Specifically, Truelove and Nugent (2020) found that environmental self-identity positively mediated the relationship between changes in straw use and changes in other self-reported behaviors, while Xu et al. (2018) found that environmental self-identity did not mediate the relationship between changes in self-reported waste separation behavior and changes in other PEBs. Complicating matters, Truelove et al. (2016) found that environmental self-identity had a (negative) indirect effect from recycling an item to environmental policy support for Democrats, but not other political groups.

### Negative Spillover

The main mechanism proposed to underlie negative PEB spillover is moral licensing (Truelove et al., 2014), as PEB is a type of moral behavior (Stern, 2000; Steg et al., 2005). According to moral licensing theory (Merritt et al., 2010; Blanken et al., 2015), when people perform an initial moral behavior, they feel released from moral constraints and are less motivated to act when given an opportunity to perform a subsequent moral behavior.

A handful of studies has assessed the role of guilt in PEB spillover under the assumption that performance of an initial PEB will reduce feelings of guilt (in line with moral licensing) and lead to a reduced likelihood of additional PEBs (Truelove et al., 2014). Previous research has found that guilt positively influences PEB (Mallett, 2012; Harth et al., 2013). However, evidence for guilt’s role in PEB spillover is mixed, with some finding guilt as a mechanism underlying negative spillover from prior PEB reminders to policy support (Lacasse, 2016, Study 1) and some finding no spillover effects through guilt from prior PEB reminders to policy support (Lacasse, 2016, Study 2), from observed recycling behavior to policy support (Truelove et al., 2016), from self-reported straw use to self-reported PEB performance or policy support (Truelove and Nugent, 2020), or from self-reported waste separation behavior to other PEBs (Xu et al., 2018).

### Aims of the Current Paper

The present studies were designed to accomplish four primary goals. Our first goal is to test a fuller model of PEB spillover to examine downstream effects of reminders of past behaviors. As described above, previous spillover research has typically referred to the past behavior as the initial PEB in the spillover sequence, testing whether making past pro-environmental actions salient spills over to later PEBs or policy support. We could find no research that extends this model to test the effect of past behavior reminders (PEB1) on behavior performance (PEB2) and then to future behavior intentions in the same study (PEB3; c.f. Lauren et al., 2016). Furthermore, we assess the hypothesized mechanisms underlying spillover at two time points: after the behavior reminders and again after the PEB performance measure, testing the process variables downstream. Knowing how past behavior reminders affect immediate behavior and process variables as well as future behavioral intentions can shed further light on the theorized mechanisms underlying spillover.

Second, we seek to advance our understanding of the role of guilt and identity in the spillover sequence. Though several researchers have proposed that guilt and environmental self-identity underlie spillover effects, as far as we know, very few studies have simultaneously evaluated the role of guilt and environmental self-identity in spillover from PEB reminders to additional PEBs (i.e., Lacasse, 2016) and only a handful have investigated the role of environmental self-identity and guilt in spillover from PEB performance to additional PEBs (Truelove et al., 2016; Xu et al., 2018; Truelove and Nugent, 2020). We assess the ability of environmental self-identity and guilt to act as mediators of both the prior PEB reminder-PEB performance relationship and the PEB performance-PEB intention relationship simultaneously in the same study (Study 2), which will allow for more controlled comparisons than that gained from comparing different studies utilizing different samples and methodologies.

Third, we aim to compare the effects of a prior PEB reminder to a reminder of environmentally unfriendly behavior, as well as to a true control condition in which perception of prior behavior is not manipulated. Previous research has typically used a prior PEB manipulation that involves checking many or few behaviors in a list based on instructions leading participants into a many prior PEB group or few prior PEB group (Van der Werff et al., 2014b; Lacasse, 2016; Lauren et al., 2019). We conduct a conceptual replication of this research by testing a novel manipulation that involves conditions where participants are asked to write about instances of previously performing environmentally friendly behaviors or environmentally harmful behaviors. Additionally, our use of a true control condition is in response to recent calls to do so in spillover research (Lacasse, 2016; Lauren et al., 2019). Comparing experimental interventions to a true control group has important implications for policy makers who need to know how their programs compare to no intervention alternatives. As far as we know, these studies will be the first to simultaneously include a true control group in the prior PEB reminder manipulation, measure actual secondary PEB performance, assess the indirect effects of environmental self-identity and guilt, and test spillover from prior PEB reminders to downstream PEB intentions.

Our fourth goal is to test PEB spillover to different types of energy-saving behaviors. Research has shown that the extent of spillover depends on the nature of the second PEB (Maki et al., 2019), but little research has investigated spillover onto different classes of behavior in the same study (c.f. Lauren et al., 2019). Much work has been done to categorize and classify PEBs (Gardner and Stern, 2008; Laitner et al., 2009; Karlin et al., 2014; Truelove and Gillis, 2018), with the simplest classification scheme of energy behaviors differentiating between curtailment behaviors (PEBs that involve reducing the use of energy-consuming products) and efficiency upgrades (PEBs that involve purchasing more energy efficient appliances and products). Curtailment PEBs are repeated actions that are low-cost or no cost, while efficiency upgrade PEBs are one-time, high-cost actions. Efficiency upgrade behaviors are more related to demographics like income and home ownership, whereas curtailment behaviors more related to values and attitudes (Karlin et al., 2014). No research could be located that evaluated PEB spillover to curtailment versus efficiency upgrade energy behaviors, though Lanzini and Thøgersen (2014) found evidence that positive spillover from “green” purchase behavior to other pro-environmental behaviors was most likely for low-cost secondary behaviors, suggesting that positive spillover to curtailment PEBs would be more likely than to efficiency upgrade PEBs. Furthermore, theory suggests that difficult secondary behaviors (e.g., efficiency upgrades) would lead to no spillover or even negative spillover as those faced with a difficult second task may be more likely to rely on their previous moral good deed as a license to refuse the difficult task (Truelove et al., 2014).

### Hypotheses

Participants in our studies completed a novel reminder of past behavior with feedback that they are an environmentally friendly person (prior PEB condition) or feedback that they are an environmentally unfriendly person [prior anti-environmental behavior (AEB) condition] or a control condition with no reminder of past behavior or feedback. Participants then completed measures of environmental self-identity and guilt and were given an opportunity to perform an actual PEB (help an environmental organization by alphabetizing a mailing list of potential new members). In Study 2 only, participants again completed measures of environmental self-identity and guilt. Finally, participants completed scales assessing intention to perform curtailment energy behaviors (Study 1 and 2) and energy efficiency upgrades (Study 1 only).

**FIGURE 1:**
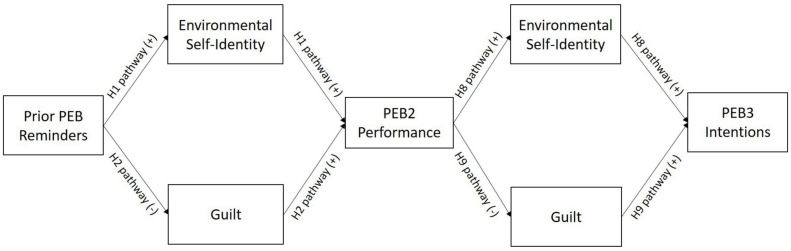
Theoretical spillover model. PEB, pro-environmental behavior. Only adjacent direct paths are shown in figure, though all downstream direct and indirect paths are theorized in the model.

In line with theory and the previous research findings outlined above, we forward the following hypotheses, based on the model in Figure 1.

#### Spillover From Behavior Reminders to PEB2 Performance

• Positive spillover via environmental self-identity (H1): Reminders of past PEBs will positively and indirectly affect PEB2 performance through increasing environmental self-identity compared to reminders of past AEBs and the control condition.
• Negative spillover via guilt (H2): Reminders of past PEBs will negatively and indirectly affect PEB2 performance through decreasing guilt compared to reminders of past AEBs and the control condition.
• Overall spillover (H3): Overall spillover effects of prior PEB reminders on PEB2 will be small and non-significant due to the competing paths above as found by Lacasse (2016).

#### Spillover From Behavior Reminders to PEB3 Intention

• Positive spillover via environmental self-identity (H4): Reminders of past PEBs will positively and indirectly affect PEB3 intentions through increasing environmental self-identity and PEB2 compared to reminders of past AEBs and the control condition.
• Negative spillover via guilt (H5): Reminders of past PEBs will negatively and indirectly affect PEB3 intentions through decreasing guilt and PEB2 performance compared to reminders of past AEBs and the control condition.
• Overall spillover (H6): Overall spillover effects from prior PEB reminders on PEB3 intentions will be small and non-significant due to the competing paths above.
• Spillover to curtailment vs. efficiency intentions (H7): Spillover effects will be larger for curtailment than efficiency intentions as efficiency intentions relate more closely to demographics than attitudinal variables (Karlin et al., 2014).

#### Spillover From PEB2 Performance to PEB3 Intention

• Positive spillover (H8): PEB2 performance will positively and indirectly affect PEB3 intentions through increasing environmental self-identity.
• Negative spillover (H9): PEB2 performance will negatively and indirectly affect PEB3 intentions through decreasing guilt.
• Overall spillover (H10): Overall spillover effects of PEB2 performance on PEB3 intentions will be small and non-significant due to the competing paths above.

## Study 1

### Method

#### Participants

Participants were recruited from Amazon’s Mechanical Turk (MTurk) worker pool with the only requirement that they are U.S. residents. Participants were paid $1.00 in exchange for completing the survey. Four hundred and forty-eight participants completed the survey. Seventy-one participants were removed from the analysis for not writing about their previous PEB in the prior behavior list (*n* = 29), expressing suspicion about the feedback (*n* = 34), missing one of the two attention checks (*n* = 6), or failing to complete more than half of the measures in the model (*n* = 2), leaving a final sample of 377 participants (206 men, 166 women, 5 gender missing). The participants were predominately White (78%) with 10% of the sample identifying as Black or African American, and ages ranged from 20 to 74 years. In terms of political affiliation, the sample was split among Democrats (46%), Republicans (24%), and other (29%).

#### Procedure

The study procedures were approved by the Institutional Review Board at the first author’s university. After agreeing to an on-line consent, participants completed basic demographic questions. Next participants were randomly assigned to either one of the experimental conditions (prior PEB or prior AEB) or the control condition. In the experimental conditions, participants were asked to review a list of six behaviors and if the behavior was one they have done regularly in the past, then to briefly describe an instance in which they performed the behavior. In the prior PEB condition, the list of behaviors were pro-environmental behaviors (e.g., recycle paper, glass, and plastic; turn the lights off when you leave a room), but in the prior AEB condition, the list of behaviors consisted of environmentally harmful behaviors (e.g., commute by driving, use plastic bags instead of reusable bags for shopping). The lists were designed so that most participants would have performed the behaviors in the past and would be able to write about these instances to make the behavior performance salient. After writing about their behavior in the experimental conditions, participants received feedback reinforcing the condition (Lacasse, 2016, Study 2). In the prior PEB condition, participants were told that their results revealed they were very helpful to the environment and care deeply about environmental issues. In the prior AEB condition, participants were told that their results revealed they were very damaging to the environment and that they don’t really care about environmental protection. Participants in the control condition did not receive the behavior list or feedback (see Supplementary Information).

Next all participants completed the six-item environmental self-identity scale (Whitmarsh and O’Neill, 2010) and a shortened version of the Positive and Negative Affect Schedule (PANAS) (Watson et al., 1988; Thompson, 2007) to mask an item assessing guilt (see Supplementary Information). Participants indicated how guilty they felt at the present moment on a scale from (1) very slightly or not at all to 5 (extremely). Participants also completed additional measures not analyzed in this manuscript.

Next participants were given an opportunity to perform a PEB (PEB2), using a new measure designed for this study. Participants were presented with a cover story describing that the researchers were working with an environmental organization focusing on climate change. Participants were asked whether they would be willing to volunteer to help the environmental organization by alphabetizing a list of individuals’ names for a mailing list. The task was described as taking approximately 1 min and participants were told that their payment in Mturk was not related to whether they agreed to help the environmental organization. Participants who agreed were presented with 10 names and addresses to alphabetize. All participants who agreed actually performed the task. We view the measure as an indicator of donating time to help an environmental organization, much like donating money to help an environmental organization, which has been used as a measure of PEB in several spillover studies (Carrico et al., 2018; Brügger and Höchli, 2019; Eby et al., 2019). Additionally, the measure is an extension of previous spillover research that asked participants to hypothetically allocate volunteer time among charities including pro-environmental organizations (Margetts and Kashima, 2017).

Then all participants completed a questionnaire assessing their intention to perform a list of PEBs in the next 6 months (PEB3 intention). Twelve of the PEBs were curtailment behaviors and participants indicated their likelihood to perform the behavior on a scale from 1 (extremely unlikely to do it) to 9 (extremely likely to do it). Eight of the questions assessed intention to perform efficiency upgrades (e.g., add home insulation to attic), so an additional response option of 10 (I have already done this behavior) was included in the response scale for these items. Eight additional PEBs, not directly related to energy use (e.g., compost kitchen waste) were included in the list, but are not analyzed in the present paper due to the focus on energy-related behaviors. Finally, participants were presented with a written debriefing.

### Results

We conducted a randomization check, which ensured that the randomly assigned groups did not differ on key demographic variables including age, gender, race, conservatism, political party, or race (see Supplementary Information).

Participants were relatively evenly split between conditions: Prior PEB (32%), Prior AEB (28%), and Control (40%). Thirty-four percent of participants agreed to perform PEB2 (alphabetize the mailing list for the environmental organization), while 66% did not agree to perform PEB2.

We created a mean score of the environmental self-identity scale items (α = 0.764, *M* = 5.158, *SD* = 1.176), with three of the items reverse-scored. We also created a mean curtailment intention scale (α = 0.847, *M* = 6.071, *SD* = 1.640) and a mean efficiency upgrade intention scale (α = 0.949, *M* = 4.507, *SD* = 2.468). Note that for the efficiency upgrade intention scale, if the participant had already completed the efficiency upgrade, it would not make sense for them to intend to complete it again in the near future, so their answers to that item were coded as missing and their mean score was calculated with all the other items in the scale. Responses to the guilt item were severely skewed [76% of all responses were (1) very slightly or not at all]. Thus, we dichotomized the guilt item such that scores of 1 (very slightly or not at all) were coded as 0 and all other responses were coded as 1 (at least a little guilt).

The variables of interest significantly, positively correlated with each other, except for guilt, which was not associated with PEB3 curtailment intentions, marginally associated with PEB3 efficiency intentions and PEB2 performance, and negatively associated with environmental self-identity (see Correlation Matrix in Supplementary Information).

#### Spillover From Past Behavior Reminders to PEB2

The hypotheses related to spillover from past behavior reminders to PEB2 performance (i.e., H1, H2, and H3) were tested using path analyses run in MPlus version 7.4 using WLSMV as the estimator with theta parameterization with 10000 bootstrap draws. The theorized model was run twice, once with the prior PEB group as the reference group for the experimental condition to allow for comparisons with previous research in the area and once with the control group as the reference group for the experimental condition to test the effects of the prior PEB and prior AEB conditions compared to a true control group. All downstream direct and indirect effects were modeled. Because the models were saturated, fit statistics were not computed (West et al., 2012).

**FIGURE 2:**
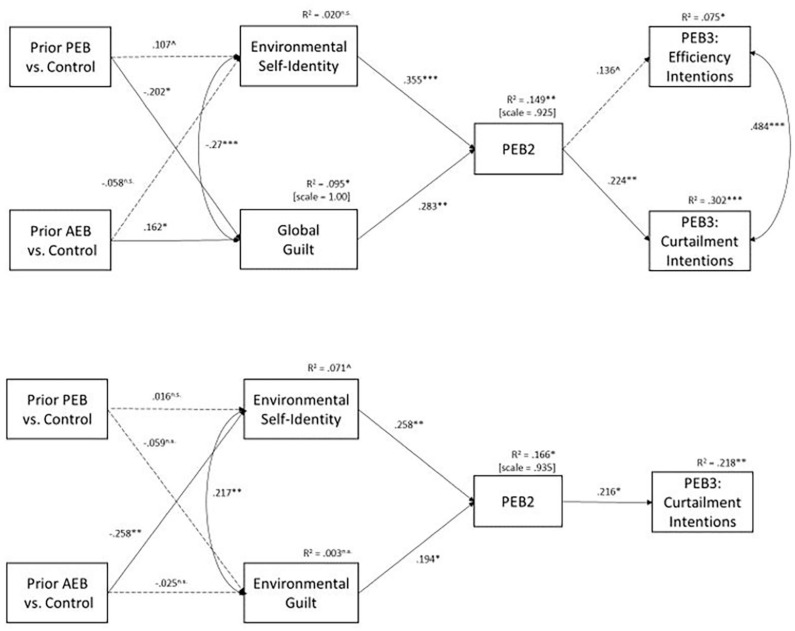
Results of direct effects in model testing spillover from prior behavior reminders to PEB2 performance, Study 1 **(top panel)** and Study 2 **(bottom panel).** PEB, pro-environmental behavior. Dashed lines represent paths with *p* > 0.05. n.s., non-significant. Only major theorized direct paths are shown in figure, though all indirect and direct paths are modeled. ***p < 0.001; **p < 0.01; *p < 0.05; ^*p* < 0.10.

First, we examined the direct effects in the hypothesized model (Figure 2 top panel, Table 1, and See Supplementary Figure with prior PEB group as comparison group). The prior PEB condition led to significantly higher (compared to the prior AEB condition) and marginally higher (compared to the control condition) levels of environmental self-identity as well as significantly lower levels of guilt (compared to the prior AEB and control conditions). The prior AEB condition also increased guilty feelings compared to the control condition. Environmental self-identity and guilt feelings were, in turn, both positively related to PEB2 performance. PEB2 performance was positively related to PEB3 curtailment intentions and marginally related to PEB3 efficiency intentions.

**TABLE 1:** Standardized direct and indirect effects on PEB2 performance.

	Study 1			Study 2		
Effect	Parameter estimate	95% CI	95% CI	Parameter estimate	95% CI	95% CI
		LL	UL		LL	UL
**Prior PEB vs. Control Condition**						
Total effect	–0.057	–0.201	0.089	0.010	–0.201	0.225
Total indirect	–0.019	–0.097	0.041	–0.007	–0.074	0.058
Via identity	0.038^	–0.001	0.086	0.004	–0.046	0.052
Via guilt	−0.057^	–0.136	–0.007	–0.011	–0.055	0.025
Direct	–0.038	–0.181	0.110	0.018	–0.187	0.223
**Prior AEB vs. Control Condition**						
Total effect	–0.072	–0.215	0.073	−0.208^	–0.408	0.010
Total indirect	0.025	–0.033	0.091	−0.072^	–0.167	0.004
Via identity	–0.020	–0.063	0.018	−0.067^	–0.154	–0.010
Via guilt	0.046^	0.004	0.101	–0.005	–0.050	0.035
Direct	–0.097	–0.214	0.049	–0.137	–0.332	0.077
**Prior AEB vs. Prior PEB Condition^#^**						
Total effect	–0.017	–0.170	0.135	−0.218*	–0.422	–0.001
Total indirect	0.044	–0.042	0.144	–0.064	–0.157	0.009
Via identity	−0.057*	–0.109	–0.016	−0.071*	–0.153	–0.014
Via guilt	0.100*	0.028	0.197	0.006	–0.031	0.044
Direct	–0.061	–0.233	0.109	–0.154	–0.357	0.074
Identity direct effect	0.355***	0.233	0.476	0.258**	0.067	0.457
Guilt direct effect	0.283*	0.089	0.475	0.194*	0.011	0.368

^#^The Prior PEB vs. Prior PEB condition comparison was run in a separate regression equation with the prior PEB condition as the comparison group. Prior AEB, anti-environmental behavior; PEB, pro-environmental behavior reminder. Study 2 Identity and Guilt refer to Time 1 measurements. Study 1 Guilt is operationalized as global guilt (binary) and Study 2 Guilt is operationalized as environmental guilt. Identity is operationalized as environmental self-identity in both studies. ***p < 0.001; **p < 0.01; *p < 0.05; ^*p* < 0.10.

We next examined the indirect effects of the prior behavior reminders on PEB2 performance (Table 1). The prior PEB condition (compared to the prior AEB condition) had a significant positive specific indirect effect on PEB2 through increasing environmental self-identity (supporting H1), and a significant negative specific indirect effect on PEB2 through guilt (supporting H2). These two specific indirect effects were in the opposite direction and effectively canceled each other out, leading to no significant total indirect effect of condition on PEB2. Thus, there was no overall spillover effect from prior behavior reminders to PEB2 when comparing the prior PEB to the prior AEB condition, supporting H3.

When comparing the prior PEB condition to the control condition, there was a marginally significant positive specific indirect effect through environmental self-identity and a marginally significant negative specific indirect effect through guilt, providing no support for H1 or H2. The total indirect effect was non-significant, indicating no spillover when comparing the prior PEB condition to the control condition, supporting H3. Overall, the prior behavior reminders seem to create individual spillover pathways by increasing environmental self-identity and decreasing guilt, thereby creating no overall spillover between the prior behavior reminders and PEB2 performance.

#### Spillover From Past Behavior Reminders to PEB3 Intention

Next, we examined the indirect effects of prior behavior reminders on PEB3 curtailment intentions and several specific indirect effects were revealed (left side of Table 2). When comparing the prior PEB to the control condition, there was a marginally significant positive specific indirect effect on curtailment intentions through environmental self-identity, though no other effects approached significance. When comparing the prior PEB condition to the prior AEB condition, there was a significant, positive specific indirect effect through environmental self-identity and a marginally significant positive specific indirect effect through environmental self-identity via PEB2 performance. Additionally, there was a marginally significant negative indirect effect through guilt via PEB2 performance. The cumulative effect of the opposing specific, indirect effects, was no overall indirect effect of PEB reminders on PEB3 curtailment intentions, supporting H6.

**TABLE 2:** Standardized direct and indirect effects on PEB3 curtailment intentions.

	Study 1			Study 2		
Effect	Parameter estimate	95% CI LL	95% CI UL	Parameter estimate	95% CI LL	95% CI UL
**PEB2**						
Direct effect	0.224**	0.088	0.364	0.216*	0.022	0.412
**Prior PEB vs. Control Condition**						
Total effect	0.000	–0.107	0.107	–0.027	–0.193	0.140
Total indirect	0.026	–0.045	0.097	–0.011	–0.103	0.075
Via PEB2	–0.009	–0.045	0.026	0.004	–0.045	0.062
Via identity	0.049^	–0.002	0.103	0.002	–0.032	0.037
Via guilt	–0.009	–0.048	0.022	–0.016	–0.072	0.034
Via identity and PEB2	0.008	0.000	0.022	0.001	–0.012	0.013
Via guilt and PEB2	–0.013	–0.036	–0.001	–0.002	–0.014	0.006
Direct	–0.027	–0.121	0.067	–0.015	–0.171	0.143
**Prior AEB vs. Control Condition**						
Total effect	–0.016	–0.133	0.100	–0.116	–0.291	0.066
Total indirect	–0.035	–0.107	0.036	–0.092	–0.224	0.020
Via PEB2	–0.022	–0.065	0.011	–0.030	–0.094	0.018
Via identity	–0.026	0.078	0.023	–0.040	–0.117	0.012
Via guilt	0.008	–0.017	0.040	–0.007	–0.068	0.047
Via identity and PEB2	–0.005	–0.016	0.004	–0.014	–0.047	0.000
Via guilt and PEB2	0.01	0.001	0.027	–0.001	–0.013	0.009
Direct	0.018	–0.091	0.129	–0.024	–0.192	0.152
**Prior AEB vs. Prior PEB Condition^#^**						
Total effect	–0.016	–0.135	0.105	–0.089	–0.259	0.088
Total indirect	–0.060	–0.142	0.020	–0.080	–0.196	0.017
Via PEB2	–0.014	–0.061	0.024	–0.033	–0.106	0.016
Via identity	−0.073**	–0.130	–0.022	–0.042	–0.119	0.014
Via guilt	0.016	–0.036	0.076	0.009	–0.040	0.057
Via identity and PEB2	−0.013^	–0.028	–0.003	–0.015	–0.046	–0.001
Via guilt and PEB2	0.022^	0.005	0.054	0.001	–0.008	0.011
Direct	0.045	–0.076	0.165	–0.009	–0.164	0.158
**Identity**						
Total effect	0.533***	0.451	0.611	0.209*	0.022	0.385
Total indirect (Via PEB2)	0.079**	0.030	0.141	0.056	0.002	0.151
Direct	0.453***	0.353	0.548	0.154	–0.062	0.340
**Guilt**						
Total effect	0.110	–0.025	0.252	0.317***	0.164	0.472
Total indirect (Via PEB2)	0.063*	0.014	0.136	0.042	–0.002	0.110
Direct	0.047	–0.100	0.194	0.275**	0.118	0.431

^#^The Prior PEB vs. Prior PEB condition comparison was run in a separate regression equation with the prior PEB condition as the comparison group.

***p < 0.001; **p < 0.01; *p < 0.05; ^*p* < 0.10.

*Prior AEB, anti-environmental behavior; PEB, pro-environmental behavior reminder. Study 2 Identity and Guilt refer to Time 1 measurements. Study 1 Guilt is operationalized as global guilt (binary) and Study 2 Guilt is operationalized as environmental guilt. Identity is operationalized as environmental self-identity in both studies.*

Next, we examined the indirect effects of prior behavior reminders on PEB3 efficiency upgrade intentions (Table 3). None of the total indirect nor specific indirect effects from prior behavior reminders to efficiency upgrade intentions were significant no matter the comparison group, failing to support H4, and H5, but in support of H6 (no total indirect effect). Thus, prior behavior reminders did not spill over to PEB3 efficiency intentions and the spillover pathways observed were larger for curtailment intentions than efficiency upgrade intentions, in support of H7.

**TABLE 3:** Standardized direct and indirect effects on PEB3 efficiency upgrade intentions, Study 1.

Effect	Parameter estimate	95% CI	95% CI
		LL	UL
**PEB2**			
Direct effect	0.136^	–0.017	0.283
**Prior PEB vs. Control Condition**			
Total effect	−0.121*	–0.233	–0.007
Total indirect	0.020	–0.074	0.025
Via PEB2	–0.005	–0.033	0.018
Via identity	0.015	–0.001	0.045
Via guilt	–0.027	–0.082	0.005
Via identity and PEB2	0.005	–0.001	0.015
Via guilt and PEB2	–0.008	–0.025	0.001
Direct	−0.102^	–0.220	0.022
**Prior AEB vs. Control Condition**			
Total effect	–0.082	–0.194	0.028
Total indirect	0.004	–0.04	0.053
Via PEB2	–0.013	–0.045	0.008
Via identity	0.008	–0.031	0.008
Via guilt	0.022	–0.004	0.063
Via identity and PEB2	–0.003	–0.011	0.003
Via guilt and PEB2	0.006	–0.001	0.018
Direct	–0.086	–0.197	0.023
**Prior AEB vs. Prior PEB Condition^#^**			
Total effect	0.034	–0.083	0.151
Total indirect	0.022	–0.036	0.096
Via PEB2	–0.008	–0.040	0.018
Via identity	–0.023	–0.058	–0.001
Via guilt	0.047	–0.009	0.125
Via identity and PEB2	–0.008	–0.020	0.001
Via guilt and PEB2	0.014	–0.002	0.036
Direct	0.012	–0.120	0.136
**Identity**			
Total effect	0.191**	0.076	0.304
Total indirect (Via PEB2)	0.048^	–0.006	0.106
Direct	0.143*	0.014	0.274
**Guilt**			
Total effect	0.173*	0.022	0.329
Total indirect (Via PEB2)	0.039	–0.005	0.097
Direct	0.135	–0.027	0.303

^#^The Prior PEB vs. Prior PEB condition comparison was run in a separate regression equation with the prior PEB condition as the comparison and is included here for ease of presentation.

**p < 0.01; *p < 0.05; ^*p* < 0.10.

*Prior AEB, anti-environmental behavior; PEB, pro-environmental behavior reminder. Guilt is operationalized as global guilt (binary). Identity is operationalized as environmental self-identity.*

### Discussion

Study 1 provided some evidence of spillover effects from reminders of prior environmental behavior to PEB2 performance. Specifically, we found evidence of competing positive and negative spillover pathways when comparing the reminder of prior PEB condition to the reminder of prior AEB conditions. Reminding people of past PEB led to positive spillover by increasing environmental self-identity, but it also led to negative spillover by reducing guilt. These effects were less strong, and only marginally significant, when comparing the reminder of prior PEB condition with the true control condition. Across all comparisons and in line with Lacasse (2016), the net effect of prior PEB reminders on PEB2 performance resulted in no spillover overall.

At the same time, our results show limited evidence of spillover from prior behavior reminders to downstream PEB3 intentions. For curtailment intentions, several spillover pathways were found, though the effects were quite small. Specifically, we found some evidence that prior PEB reminders indirectly affected curtailment intentions through increasing environmental self-identity and the environmental self-identity-to-PEB2 pathway and decreasing the guilt-to-PEB2 pathway, though these effects were stronger when comparing the prior PEB condition to the prior AEB condition as opposed to the true control condition. For efficiency intentions, we found no evidence of spillover effects from the prior behavior manipulation.

As the first study to explore a sequence of spillover from prior behavior reminders to PEB2 performance to PEB3 intentions, Study 1 provides initial evidence that spillover effects from prior behavior reminders may fade downstream. However, Study 1 did not assess environmental self-identity and guilt between PEB2 performance and PEB3 intentions, which would allow for a fuller test of the theoretical model in Figure 1. Additionally, Study 1′s findings that environmental self- identity effects were stronger than those of guilt could be a result of either the constructs themselves or the measurement techniques employed, as self-identity was assessed as environmental self-identity, while guilt was assessed as a global measure of guilt. Scales that more clearly link guilt with one’s prior performance of anti-environmental actions (e.g., Mallett, 2012; Bissing-Olson et al., 2016) may be even more likely to relate to PEB performance. In Study 2, we remedy both of these issues by utilizing a measure of environmental guilt and assessing environmental self-identity and guilt after the prior PEB reminder (as in Study 1) and again after the PEB2 performance (new to Study 2).

## Study 2

### Aims

Study 2 serves as a conceptual replication and extension of Study 1 using data gathered from a student sample. We utilized updated measures of environmental self-identity and environmental guilt and measured these constructs at both time periods as indicated in the hypothesized theoretical model (Figure 1), allowing us to test H8–H10. We also focused on curtailment intentions, as the Study 1 results indicated no spillover to efficiency upgrades.

### Method

#### Participants

Participants were recruited from the undergraduate psychology subject pool at a mid-sized university in the southeastern United States and participated as one option for extra credit in their psychology courses. One hundred and ninety-two participants began the survey. Twenty participants were removed from the analysis for expressing suspicion about the feedback (*n* = 7); agreeing to perform PEB1, but not actually alphabetizing the list (*n* = 10); or failing to answer at least one of the questions relating to the variables of interest (*n* = 3); leaving a final sample of 172 participants (25 men, 135 women, 12 gender missing). The participants were predominately White (73%) with 13% of the sample identifying as Black or African American and 9% identifying as Asian. Ages ranged from 18 to 50 years, with an average age of 21 years old. In terms of political affiliation, the sample was split among Democrats (40%), Republicans (21%), and other (38%). The sample size was determined based on feasibility – we collected as many participants as possible over the course of the semester based on availability in the subject pool.

#### Procedure

The study procedures were approved by the Institutional Review Board at the first author’s university and mirror those used in Study 1. After agreeing to an online consent, participants completed basic demographic questions. Next participants were randomly assigned to the same prior behavior manipulation as in Study 1.

Then all participants completed a three-item measure of environmental self-identity (Van der Werff et al., 2013) (α = 0.924, *M* = 4.928, *SD* = 1.269) and a three-item measure of environmental guilt (adapted from Bissing-Olson et al., 2016; Xu et al., 2018) (α = 0.878, *M* = 3.828, *SD* = 1.451) (see Supplementary Information). Participants also completed additional measures not analyzed in this manuscript.

Next participants were given the same opportunity to perform PEB2 as in Study 1. Then participants repeated the same measures of environmental self-identity (α = 0.935, *M* = 4.897, *SD* = 1.340) and environmental guilt (α = 0.903, *M* = 3.891, *SD* = 1.496). Finally, all participants completed the same PEB3 intention scale as in Study 1, though only the 12 curtailment intentions were analyzed in this analysis (α = 0.720, *M* = 6.075, *SD* = 1.279) (see Supplementary Information). Finally, participants were presented with a written debriefing.

### Results

We conducted a randomization check, which ensured that the randomly assigned groups did not differ on key demographic variables including age, gender, race, conservatism, political party, or race (see Supplementary Information).

Participants were relatively evenly split between conditions: Prior PEB (34%), Prior AEB (32%), and Control (34%). Forty-three percent of participants agreed to perform PEB2 (alphabetize the mailing list for the environmental organization), while 57% did not agree to perform PEB2.

The variables of interest significantly, positively correlated with each other (see Correlation Matrix in Supplementary Information), including environmental guilt, whereas in Study 1 global guilt was not positively associated with PEB3 curtailment. The correlations between environmental self-identity at Time 1 and Time 2 and environmental guilt at Time 1 and Time 2 were extremely strong (i.e., >0.84), suggesting that they are collinear and should not be entered into the same regression model simultaneously (Allen, 1997).

#### Spillover From Past Behavior Reminders to PEB2 Performance

The hypotheses related to spillover from past behavior reminders to PEB2 performance (i.e., H1, H2, and H3) were tested using the same process as in Study 1 (Figure 2 bottom panel, Table 1 right panel, and see Supplementary Information for Figure with prior PEB as comparison group). The prior PEB condition led to increases in the levels of environmental self-identity when compared to the prior AEB condition, but not the control condition. The prior PEB condition did not lead to decreases in environmental guilt (when compared to the prior AEB or control conditions). Additionally, the prior AEB condition (compared to the control condition) significantly decreased environmental self-identity levels, but did not affect environmental guilt levels. Environmental self-identity and environmental guilt both positively related to PEB2 performance. PEB2 performance was positively related to PEB3 curtailment intentions.

We next examined the indirect effects of the prior behavior reminders on PEB2 performance (right side of Table 1). When comparing the prior PEB condition to the prior AEB condition, the prior PEB condition had a significant positive specific indirect effect on PEB2 through increasing environmental self-identity (supporting H1), and a negative, though not significant, specific indirect effect on PEB2 through guilt (failing to support H2). These indirect effects acted in opposite directions leading to a non-significant overall indirect effect of condition on PEB2. Thus, there was no overall spillover effect from prior behavior reminders to PEB2 performance when comparing the prior PEB to the prior AEB condition, supporting H3. When comparing the prior PEB condition to the control condition, there were no significant specific indirect effects through environmental self-identity or environmental guilt. The total indirect effect was non-significant, indicating no spillover when comparing the prior PEB condition to the control condition, supporting H3. Overall, the prior pro-environmental behavior reminders seem to create small and non-significant spillover pathways through increasing environmental self-identity and decreasing guilt, thereby creating no overall spillover between the prior behavior reminders and PEB2 performance.

#### Spillover From Past Behavior Reminders to PEB3 Intention

Next, we examined the indirect effects of prior behavior reminders on PEB3 curtailment intentions, and none of the overall indirect effects nor specific indirect effects through PEB1 or environmental self-identity and guilt at T1 were significant (right side of Table 2) no matter the comparison group, failing to support H4 and H5, but in support of H6 (no overall spillover effect).

#### Spillover From PEB2 Performance to PEB3 Intention

**FIGURE 3:**
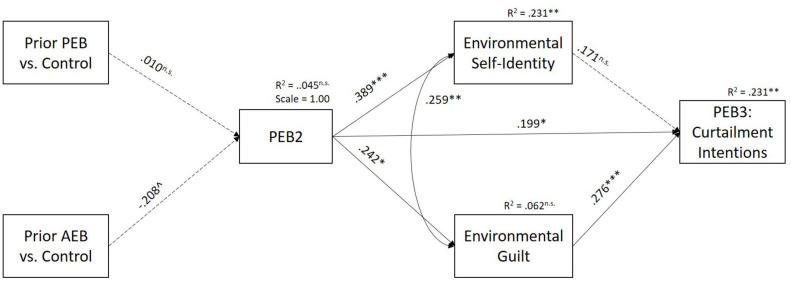
Results of hypothesized direct effects in model testing spillover from PEB2 performance to PEB3 curtailment intentions, Study 2. PEB, pro-environmental behavior. Dashed lines represent *p* > 0.05. Environmental self-identity and environmental guilt refer to Time 2 measures, which were assessed after PEB2. Only major theorized paths are shown in figure, though all indirect and direct paths are modeled. ^∗∗∗^*p* < 0.001; ^∗∗^*p* < 0.01; ^∗^*p* < 0.05; ^*p* < 0.10.

Finally, we tested the direct and indirect effects from PEB2 performance to PEB3 curtailment intentions when Time 2 environmental self-identity and environmental guilt were entered into the model instead of Time 1 environmental self-identity and guilt (Figure 3). The model also included the condition dummy variables as predictors of PEB2 performance, though the indirect effects of the prior behavior reminders on PEB3 intentions are not discussed here because they are redundant to that discussed above.

There was a significant, positive direct effect of PEB2 on PEB3 curtailment intentions (Table 4). When testing the hypothesized positive indirect path through environmental self-identity, PEB2 performance positively related to environmental self-identity, but environmental self-identity was not significantly related to PEB3 curtailment intentions, resulting in a non-significant indirect effect of PEB2 performance on PEB3 intentions through environmental self-identity, failing to support H8. When testing the hypothesized negative indirect path through guilt, PEB2 performance positively related to environmental guilt and guilt, in turn, positively related to PEB3 curtailment intentions, resulting in a significant positive indirect effect from PEB2 to PEB3 curtailment intentions through environmental guilt, which is the opposite sign as to that proposed in H9. Overall, the indirect effect of PEB2 performance on PEB3 curtailment was positive and significant, failing to support H10, that the effect would be non-significant.

**TABLE 4:** Standardized direct and indirect effects from PEB2 to PEB3 curtailment intentions, Study 2.

Effect	Parameter estimate	95% CI	95% CI
		LL	UL
**PEB2**			
Total effect	0.333***	0.139	0.515
Total indirect	0.134**	0.036	0.237
Via identity T2	0.067	–0.020	0.155
Via guilt T2	0.067*	0.013	0.136
Direct	0.199*	0.001	0.399
Identity T2 direct effect	0.171	–0.045	0.368
Guilt T2 direct effect	0.276***	0.126	0.422

***p < 0.001; **p < 0.01; *p < 0.05.

*Guilt is operationalized as environmental guilt. Identity is operationalized as environmental self-identity.*

### Discussion

Using different measures of environmental self-identity and guilt, Study 2 replicated the results of Study 1 in that when comparing reminders of prior environmental behavior to prior AEB, there were specific indirect effects from prior environmental behavior reminders to PEB2 performance that were positive through environmental self-identity and negative through guilt. When comparing reminders of PEB to the control condition, these specific indirect effects that were marginally significant in Study 1, were not significant in Study 2. Overall, regardless of the comparison group and in line with Study 1 results, Study 2 found that reminding people about prior PEB led to no total indirect effect as the negative indirect path through guilt and the positive indirect path through environmental self-identity canceled each other out, demonstrating no spillover from prior PEB reminders to PEB2 performance, in line with Lacasse (2016).

Unlike Study 1, which found some limited evidence of spillover pathways from prior PEB reminders to PEB3 curtailment intentions through environmental self-identity and guilt, Study 2 found no evidence of spillover pathways from reminders of prior PEB to PEB3 curtailment intentions. Taken together, the paper shows little support for the idea of downstream spillover from reminders of prior PEB to behavioral intentions beyond an initial secondary behavior.

As an advance to Study 1, Study 2 was designed to also be able to test the mechanisms underlying spillover from PEB2 performance to PEB3 curtailment intentions. Study 2 showed that when investigating spillover from PEB2 performance to PEB3 intention, the hypothesized positive spillover pathway through environmental self-identity was not found as environmental self-identity at Time 2 did not relate to PEB3 curtailment intentions when controlling for environmental guilt at Time 1 and the prior behavior manipulation. Instead, a positive spillover path via environmental guilt was found, which is in the opposite direction to that hypothesized. Specifically, although it was expected that performance of PEB2 would lead to reduced levels of guilt about prior AEB, this was not the case. PEB2 performance actually led to more environmental guilt. It could be that performance of a single PEB, rather than being reminded of a performance of many PEBs, leads to acknowledgment of other times PEB was not performed leading to increasing guilt levels. Future research should explore the relationship between feelings of global guilt and environmental guilt following PEB performance.

## General Discussion

In general, we found partial support for the model proposed in Figure 1. Reminders of prior PEB can spill over to PEB2 performance and PEB3 intentions and PEB2 performance can spill over to PEB3 intentions via influencing environmental self-identity and guilt. However, our results make clear that the strength of these effects are dependent on several factors including whether the prior behavior manipulation includes a true control group, the way that environmental self-identity and guilt are measured, and the way PEB is operationalized.

### Prior Behavior Manipulation Comparison Group

This paper utilized a novel manipulation as a reminder of prior environmental behavior, namely having participants write about their prior PEB or prior AEB and labeling them as a person who is either pro-environmental or anti-environmental, respectively, or assigning them to a no intervention group. This manipulation builds on previous prior PEB reminders used in research that ask people to complete checklists of many or few pro-environmental behaviors they perform (Cornelissen et al., 2008; Van der Werff et al., 2014a; Lacasse, 2016). Lacasse (2016) found that labeling participants in the many PEB condition as environmentalists helped reduce the negative effect of the manipulation on guilt levels, and we adopted the same procedure in our study. However, our prior PEB manipulation affected both guilt and environmental self-identity levels, though the strength of the effects depended on the comparison group. Relative to the prior AEB condition, the prior PEB manipulation increased environmental self-identity in both Studies and reduced guilt in Study 1, mirroring the results of Lacasse (2016). When compared to the control group, the prior PEB group did not demonstrate increased environmental self-identity in Study 1 or Study 2, but did show reduced guilt levels as in Study 1. Thus, our manipulations of prior PEB did not consistently and uniquely elicit increases in environmental self-identity and decreases in guilt as we had expected. As it stands, we know that prior PEB reminders often elicit environmental self-identity, and sometimes elicit guilt. Messages that can isolate environmental self-identity or guilt or encourage increases in both may be best at generating positive spillover. Effective design of interventions that increase environmental self-identity, but do not reduce guilt, should be a focus of future research. If reminders of prior PEB are continued to be used in spillover research, further articulation of the theorized effects of these manipulations are needed.

Considering that most previous work has used the checklist procedure whereby participants check few or many previous PEBs based on their checklist instructions and few studies use a true control group (Van der Werff et al., 2014b, Study 3), much of what is known about the effect of a reminder of prior PEB is in comparison to a reminder of performance of fewer PEBs. In our studies, spillover effects from prior PEB reminders to PEB performance and PEB curtailment intentions were consistently larger when compared to a prior AEB reminder group than a true control group. This suggests caution in interpreting spillover effects when true control groups are not included. From a practical standpoint, we are concerned not only with those who are targeted by the intervention and change (versus those who do not change), but also those who are not targeted at all. Including non-active control groups can allow for comparisons to be made about the effectiveness of the appeal on PEBs to determine which type of appeals, if any, are best. This would result in more efficient use of limited resources for environmental campaigns.

### Conceptualization of Identity and Guilt

The relationships between the prior behavior manipulation and identity and guilt are further complicated by the measurement of identity and guilt. Studies 1 and 2 used similar measures of environmental self-identity, but quite different measures of guilt. In Study 1, guilt was measured with a single-item measure assessing feelings of global guilt in the present moment, while in Study 2, guilt was measured with several items assessing feelings of guilt over prior environmentally unfriendly behavior. The conceptualization of identity as a specific environmental self-identity and guilt as a global emotion was potentially problematic along similar lines to the compatibility principle (Ajzen and Fishbein, 2005), so we opted to update the measure of guilt in Study 2. Interestingly, the measure of environmental guilt was not significantly affected by the prior behavior manipulation in Study 2, no matter the comparison group, in contrast to Study 1 when prior PEB reminders led to lower global guilt levels compared to both the control and prior AEB reminders conditions. Furthermore, environmental self-identity was positively correlated with environmental guilt (Study 2) and negatively correlated with global guilt (Study 1). This has major implications for spillover theory and may be able to explain some conflicting results from previous research. It could be that global guilt is more transient than environmental guilt and attempts to assess long term moral licensing in relation to PEB spillover should focus on environmental guilt, not global guilt.

### PEB Operationalization

Previous research has generally investigated spillover from one PEB to one or several other PEBs. Rarely, if ever, have researchers investigated the spillover of one PEB to a second PEB and then a third PEB in a sequence. In this work, we found some evidence of spillover pathways from a prior PEB reminder to an actual PEB2 performance as well as limited evidence of spillover from a prior PEB reminder to PEB3 intentions further downstream, though no overall spillover was found from prior PEB reminders to either of these other PEBs. However, we did find that actual performance of PEB2 spilled over to positively influence PEB3 intentions. Our results support the finding that the type of PEB (e.g., behavior, intention) influences the likelihood of spillover effects (Maki et al., 2019). In line with Maki et al. (2019), we found stronger spillover effects when the initial behavior was measured as an actual behavior rather than a reminder of prior PEB and also when the secondary behavior was measured as intentions rather than behavior.

Our results also provide evidence that even when measured in the same way, different types of PEB may be more likely to follow from spillover. In Study 1, we assessed spillover to both curtailment intentions and efficiency upgrade intentions. The differing pattern of results we obtained when comparing spillover to curtailment versus efficiency intentions may help explain some of the discrepancy found in previous spillover research. The curtailment versus efficiency dichotomy is related to difficulty (Truelove and Gillis, 2018), and some work has theorized that difficulty plays a role in whether spillover occurs (Maki et al., 2019). We found that a prior PEB reminder manipulation was associated with some positive indirect spillover pathways to the relatively easy curtailment intentions, while the prior behavior manipulation did not spill over to the relatively difficult efficiency intentions. This fits with work showing that curtailment behaviors are more likely than efficiency behaviors to be influenced by attitudinal variables (Karlin et al., 2014). However, considering that efficiency upgrades are among the most impactful PEBs (Gardner and Stern, 2008), there is a real practical need to understand whether prior PEB reminders or curtailment PEBs can spill over to efficiency upgrades.

### Limitations and Suggestions for Future Research

Our project has several limitations. First, our studies were each conducted in one session. Considering that positive spillover is expected to build over time as each behavior performance reinforces environmental identity (Truelove et al., 2014), our findings related to identity as a mechanism underlying spillover need further exploration. This is especially important in light of the strong correlations we obtained between Time 1 and Time 2 environmental self-identity and environmental guilt, such that they were not able to be entered into the same regression equation to fully test Figure 1 in one study. As pointed out by a reviewer, these strong correlations could indicate that participants aim to be consistent in their responses over time, limiting our confidence in our findings related to Time 2 environmental self-identity and environmental guilt. Specifically, our finding regarding Time 2 environmental self-identity not relating to PEB3 (curtailment intentions) does not fit in line with previous research and could suggest a methodological artifact. However, these concerns are somewhat allayed by our finding that Time 2 environmental guilt did relate to curtailment intentions. Nevertheless, it is possible that people are more consistent in presentation of their self-concept than demonstration of environmental guilt such that repeated measure of self-identity versus guilt could be more prone to elicit consistency in responses. A longitudinal design (Lanzini and Thøgersen, 2014; Lauren et al., 2016; Sintov et al., 2017; Carrico et al., 2018; Xu et al., 2018; Truelove and Nugent, 2020), that allows for collecting environmental self-identity and guilt following the prior PEB manipulation in an initial session and then reassessing these constructs in a second session after a PEB had been performed for a certain period of time would help disentangle these effects. Future research should investigate the spillover sequence across multiple timepoints to allow for identity effects to develop and take hold.

Second, we focused on testing specific propositions that moral licensing is driven by guilt reduction and positive spillover is driven by environmental identity (Truelove et al., 2014), leaving other PEB spillover theories untested in this project. Specifically, although outside of the scope of the present project, our measurement of both positive and negative emotions in Study 1 lends itself to questions about the relative strength of positive versus negative emotions in driving PEB spillover. Positive emotions have been shown to relate to PEB performance (Harth et al., 2013; Ibanez et al., 2017; Rezvani et al., 2017), but only a few studies have examined the role of positive emotions in PEB spillover (Chatelain et al., 2018) and more work is needed in this area. Additionally, although the present study focused on guilt reduction as the mechanism underlying moral licensing effects, other negative emotions have been shown to relate to PEB (Harth et al., 2013; Rees et al., 2015). Furthermore, other explanations of negative PEB spillover, such as single-action bias (Weber, 1997), may implicate other negative emotions (e.g., fear) as important in PEB spillover. Future research should aim to more fully examine the role of a broader range of negative emotions as well as positive emotions in PEB spillover (Nilsson et al., 2016) and directly compare the role of negative and positive emotions as contributors to PEB spillover.

Third, a benefit of our work is that we utilize two different samples, MTurk participants and undergraduate students. However, both of our samples are convenience samples, limiting the generalizability of our findings. Additionally, the sample size in Study 2 was limited based on logistics, which could have reduced our ability to detect effects. Future research should seek to conduct spillover research on larger samples that more closely reflect the general public.

Finally, though we do measure PEB2 as actual behavior performance, our work is in line with previous research that has often relied on self-reported measures of PEB or prior PEB reminders. Future work should assess actual PEB performance, not PEB intentions or PEB reminders, for all PEBs in the sequence and should consider measuring behavioral frequency instead of binary behavior completion measures to allow for capturing a broader spectrum of PEB performance. Additionally, although our work can contribute to our understanding of spillover effects to various PEB types, research that compares multiple measurements of the same PEB in one study would be more beneficial. For example, assessing PEB2 by randomly assigning half of the participants to be given an option to perform an observed PEB and the other half being asked their intention to perform the PEB would contribute more strongly to our understanding of the relative spillover effects of an initial PEB to a secondary PEB that is measured as actual PEB performance versus intention.

## Data Availability Statement

The raw data supporting the conclusions of this article will be made available by the authors, without undue reservation.

## Ethics Statement

The studies involving human participants were reviewed and approved by University of North Florida. The patients/participants provided their written informed consent to participate in this study.

## Author Contributions

HT: conceptualization, methodology, formal analysis, investigation, writing – original draft, and project administration. AC: conceptualization and writing – review and editing. KY: conceptualization, methodology, and writing – review and editing. JW: conceptualization, formal analysis, and writing – review and editing. All authors contributed to the article and approved the submitted version.

## Conflict of Interest

The authors declare that the research was conducted in the absence of any commercial or financial relationships that could be construed as a potential conflict of interest.
