# Supplementary material for: Identity and Guilt as Mediators of Pro-environmental Spillover
Source: Front Psychol. 2021 Jun 24;12:659483. doi: 10.3389/fpsyg.2021.659483 (PMC8265758; doi:10.3389/fpsyg.2021.659483)
Supplement: Supplementary file 1 [file Data_Sheet_1.docx]

**Supplemental Information**

**Experimental Manipulation, Study 1 and Study 2**

***Prior Pro-environmental Behavior Condition***

**Behavior List**

In each line below there is a specific behavior.  If it is a behavior that you commonly engage in or have done regularly in the past, please briefly describe an instance that you performed the behavior in the corresponding space.  Otherwise, please leave the space blank.

- In summer, keep AC thermostat at 78° F or higher
- Maintain correct tire pressure on car
- Recycle paper, glass, and plastic
- Run your dishwasher when there’s a full load only
- Turn the lights off when you leave a room
- Wash clothes in warm or cold wash cycle and cold rinse cycle

**Feedback**

Based on the behavior that you perform, results show that you have

- prevented an exceptionally high amount of greenhouse gases from entering into the atmosphere.

Your behaviors are

- very helpful to the environment.

You appear to be the type of person who

- is concerned about what you can do to preserve the environment.
- has a strong commitment to environmental protection.
- cares deeply about the environment and environmental protection.

***Prior Anti-environmental Behavior Condition***

**Behavior List**
In each line below there is a specific behavior.  If it is a behavior that you commonly engage in or have done regularly in the past, please briefly describe an instance that you performed the behavior in the corresponding space.  Otherwise, please leave the space blank.

- Buy a new computer every year
- Commute by driving
- Use plastic bags instead of reusable bags for shopping
- Leave water running while soaping during shower
- Eat meat
- Keep appliances such as TV/stereo/game console in stand-by mode

**Feedback**

Based on the behavior that you perform, results show that you have

- generated an exceptionally high amount of greenhouse gases into the atmosphere.

Your behaviors are

- very damaging to the environment.

You appear to be the type of person who

- is not concerned about what you can do to preserve the environment.
- has a strong commitment to resource consumption.
- doesn't really care about the environment and environmental protection.

**Survey Measures, Study 1**

***Environmental Self-Identity (adapted from Whitmarsh and O’Neill (2010))***

Please rate your agreement with the following items.

*Strongly Disagree = 1, Disagree = 2, Slightly disagree = 3, Neither agree nor disagree = 4, Slightly Agree = 5, Agree = 6, Strongly Agree = 7*

1. I think of myself as an environmentally-friendly consumer
2. I think of myself as someone who is very concerned with environmental issues
3. I would be embarrassed to be seen as having an environmentally-friendly lifestyle (R)
4. I would not want my family and friends to think of me as someone who is concerned about environmental issues (R)
5. To engage in environmentally-friendly behavior is an important part of who I am
6. I am not the type of person oriented to engage in environmentally-friendly behavior (R)

***Guilt (Thompson (2007), adapted from Watson et al. (1988))***

Please indicate to what extent you feel this way right now, that is, at the present moment

*Very slightly, or not at all = 1, a little = 2, moderately = 3, quite a bit = 4, extremely = 5*

1. Upset
2. Hostile
3. Alert
4. Ashamed
5. Inspired
6. Nervous
7. Determined
8. Attentive
9. Afraid
10. Active
11. Guilty

**Survey Measures, Study 2**

***Environmental Self-Identity (Van der Werff et al. (2013))***

Please rate how much you agree or disagree with the following items.

*Strongly Disagree = 1, Disagree = 2, Somewhat disagree = 3, Neither agree nor disagree = 4, Somewhat Agree = 5, Agree = 6, Strongly Agree = 7*

1. Acting environmentally friendly is an important part of who I am
2. I am the type of person who acts environmentally friendly
3. I see myself as an environmentally friendly person

***Environmental Guilt (adapted from Bissing-Olsen et al. (2016) and Xu et al. (2018))***

Please rate the extent to which you experience each of the following emotions when considering your past environmentally unfriendly behaviors.

*Not at all = 1, Very Much = 7*

1. Guilt
2. Disappointment
3. Regret

**PEB2, Study 1 and Study 2**

A grassroots environmental company, Nature’s Keeper, is attempting to spread the word about small environmental tips to help reduce climate change effects. They have asked us to partner with them and get assistance from our survey respondents in alphabetizing a mailing list that they will use to send out a top 10 environmental tips newsletter.

Assisting Nature’s Keeper will help the environment and reduce climate change. If you would like to help Nature’s Keeper by alphabetizing a mailing list of 10 names, please check the “Yes” option at the bottom of the screen.

It will take approximately 1 minute to complete the tasks. Your payment [credit] will NOT be affected if you choose to complete this task or not.

If you agree to assist them,  you will find the mailing list and the instructions on the next page. After you complete the task, you will continue to the rest of the survey.

If you do not agree to assist them, you will immediately continue to the rest of the survey.

*Would you like to help the environmental organization?*

- Yes
- No

**PEB3 Curtailment Intention Scale, Study 1 and Study 2 (adapted from Lanzini & Thøgersen, 2014; Thomas et al., 2016)**

*How likely is it that you will do this behavior within the next 6 months?*

Extremely unlikely to do it = 1, extremely likely to do it = 9

1. Choose a vegetarian meal over a beef dish
2. Get a car tune-up, including air filter change
3. Move thermostat down 2 degrees in the winter
4. Recycle glass
5. Reduce highway speed from 70 to 60 mph
6. Take a shower shorter than 5 minutes
7. Turn off car if idling longer than 30 seconds
8. Turn off lights when not in use
9. Turn off the tap while brushing teeth
10. Unplug television when not in use
11. Wait until dishwasher is full before running
12. Walk/cycle instead of driving to places within 1 mile

**PEB3 Efficiency Upgrade Intention Scale, Study 1 (adapted from Lanzini & Thøgersen, 2014; Thomas et al., 2016)**

*How likely is it that you will do this behavior within the next 6 months?*

Extremely unlikely to do it = 1, extremely likely to do it = 9, I have already done this = 10 (only asked for the efficiency behaviors)

1. Add insulation material to home attic
2. Adjust water heater to no higher than 120 degrees F
3. Apply insulation to home water heater
4. Caulk/weather-strip doors and windows of home
5. Install a renewable energy system in home
6. Install an energy-efficient refrigerator
7. Replace current vehicle with a fuel-efficient vehicle
8. Switch to energy-efficient light bulbs

**Randomization Check**

***Study 1***

One-way ANOVAs in SPSS v. 27 showed that there was no effect of condition on conservatism, *F_W_* (2, 235.784) = 2.555, *p* = .080 (Welch test as homogeneity of variances assumption was violated, *p* = .023), or on age, *F* (2, 372) = 1.701, *p* = .184. Furthermore, Chi-square tests revealed that there was no relationship between condition and race, Χ^2^ (6) = 6.173, *p* = .404, between condition and gender, Χ^2^ (2) = .593, *p* = .743, or between condition and political party, Χ^2^ (4) = 8.279, *p* = .082. Thus, the randomly assigned groups did not differ on key demographic variables.

***Study 2***

One-way ANOVAs in SPSS v. 27 showed that there was no effect of condition on conservatism, *F* (2, 168) = 1.279, *p* = .281, or on age, *F* (2, 166) = 1.762, *p* = .175. Furthermore, Chi-square tests revealed that there was no relationship between condition and race, Χ^2^ (6) = 2.115, *p* = .909, between condition and gender, Χ^2^ (2) = 4.647, *p* = .098, or between condition and political party, Χ^2^ (4) = 5.612, *p* = .230. Thus, the randomly assigned groups did not differ on key demographic variables.

**Correlation Matrix**

***Study 1***

| **SI Table 1**  **Correlation Matrix, Study 1** |  |  |  |  |  |
| --- | --- | --- | --- | --- | --- |
| Variable | PEB3 Efficiency | PEB3 Curtailment | PEB2 | Identity | Guilt |
| PEB3 Efficiency | 4.507 (2.468) | .502*** | .203** | .134** | .125^ |
| PEB3 Curtailment |  | 6.071 (1.640) | .355*** | .496*** | -.037 |
| PEB2 |  |  | N/A | .275*** | .164^ |
| Identity |  |  |  | 5.158 (1.176) | -.296*** |
| Guilt |  |  |  |  | N/A |
| *Note*. Means and SDs for continuous variables on the diagonal. Study 1 Guilt is operationalized as global guilt (binary). Identity is operationalized as environmental self-identity.  ****p* < .001. ***p* < .01. **p* < .05. ^*p* < .10. | | | | | |

***Study 2***

| **SI Table 2** |  |  |  |  |  |  |
| --- | --- | --- | --- | --- | --- | --- |
| **Correlation Matrix, Study 2** | | | | | | |
| Variable | PEB3 Curtailment | PEB2 | Identity T1 | Guilt T1 | Identity T2 | Guilt T2 |
| PEB3 Curtailment | 6.075 (1.279) | .337*** | .288** | .360*** | .346*** | .378*** |
| PEB2 |  | N/A | .339*** | .243** | .435*** | .235* |
| Identity T1 |  |  | 4.928 (1.269) | .207** | .846*** | .231** |
| Guilt T1 |  |  |  | 3.828 (1.451) | .295*** | .907*** |
| Identity T2 |  |  |  |  | 4.897 (1.340) | .320*** |
| Guilt T2 |  |  |  |  |  | 3.891 (1.496) |

*Note*. Means and SDs for continuous variables on the diagonal. Study 2 Guilt is operationalized as environmental guilt. Identity is operationalized as environmental self-identity.

****p* < .001. ***p* < .01. **p* < .05. ^*p* < .10.

**Figure SI1**

*Results of direct effects in model testing spillover from prior behavior reminders to PEB2 performance with Prior PEB group as comparison group, Study 1 (top panel) and Study 2 (bottom panel)*


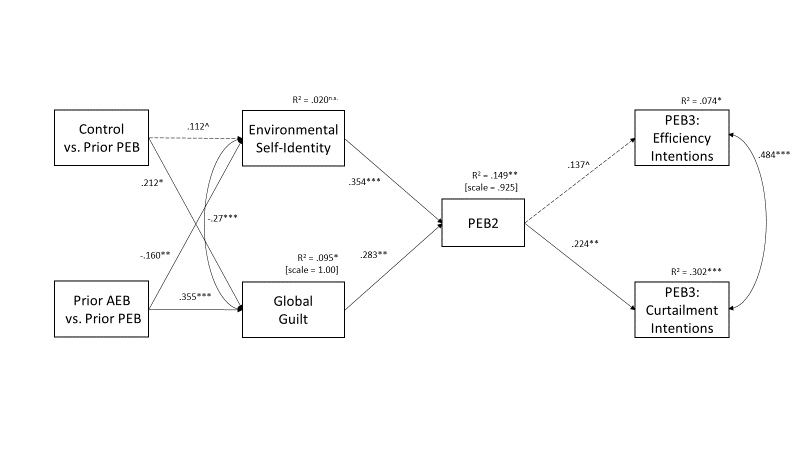


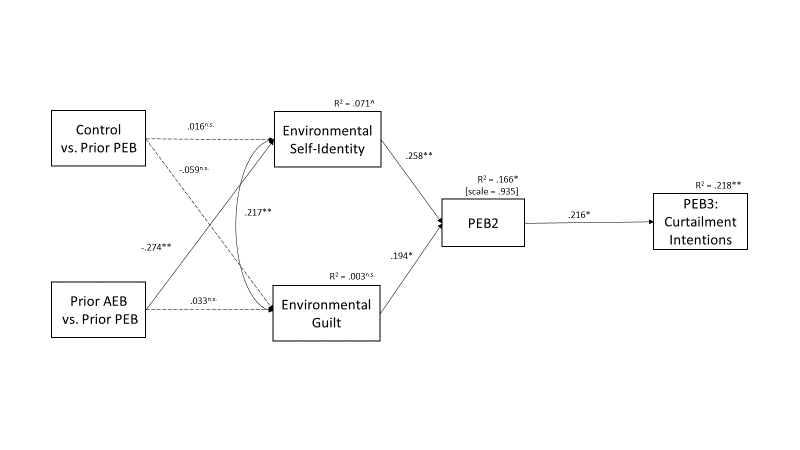


Note. PEB = Pro-environmental behavior. Dashed lines represent paths with *p* > .05. n.s. = nonsignificant.

****p* < .001. ***p* < .01. **p* < .05. ^*p* < .10.
